# Clinical parameters affect the structure and function of superficial pyramidal neurons in the adult human neocortex

**DOI:** 10.1093/braincomms/fcae351

**Published:** 2024-10-07

**Authors:** Maximilian Lenz, Pia Kruse, Amelie Eichler, Jakob Straehle, Hanna Hemeling, Phyllis Stöhr, Jürgen Beck, Andreas Vlachos

**Affiliations:** Department of Neuroanatomy, Institute of Anatomy and Cell Biology, Faculty of Medicine, University of Freiburg, 79104 Freiburg, Germany; Institute of Neuroanatomy and Cell Biology, Hannover Medical School, 30625 Hannover, Germany; Department of Neuroanatomy, Institute of Anatomy and Cell Biology, Faculty of Medicine, University of Freiburg, 79104 Freiburg, Germany; Institute of Neuroanatomy and Cell Biology, Hannover Medical School, 30625 Hannover, Germany; Department of Neuroanatomy, Institute of Anatomy and Cell Biology, Faculty of Medicine, University of Freiburg, 79104 Freiburg, Germany; Institute of Neuroanatomy and Cell Biology, Hannover Medical School, 30625 Hannover, Germany; Department of Neurosurgery, Medical Center, Faculty of Medicine, University of Freiburg, 79106 Freiburg, Germany; Center for Advanced Surgical Tissue Analysis (CAST), Faculty of Medicine, University of Freiburg, 79106 Freiburg, Germany; Department of Neuroanatomy, Institute of Anatomy and Cell Biology, Faculty of Medicine, University of Freiburg, 79104 Freiburg, Germany; Department of Neuroanatomy, Institute of Anatomy and Cell Biology, Faculty of Medicine, University of Freiburg, 79104 Freiburg, Germany; Department of Neurosurgery, Medical Center, Faculty of Medicine, University of Freiburg, 79106 Freiburg, Germany; Center for Advanced Surgical Tissue Analysis (CAST), Faculty of Medicine, University of Freiburg, 79106 Freiburg, Germany; Center for Basics in NeuroModulation (NeuroModulBasics), Faculty of Medicine, University of Freiburg, 79106 Freiburg, Germany; Department of Neuroanatomy, Institute of Anatomy and Cell Biology, Faculty of Medicine, University of Freiburg, 79104 Freiburg, Germany; Center for Advanced Surgical Tissue Analysis (CAST), Faculty of Medicine, University of Freiburg, 79106 Freiburg, Germany; Center for Basics in NeuroModulation (NeuroModulBasics), Faculty of Medicine, University of Freiburg, 79106 Freiburg, Germany; Center BrainLinks-BrainTools, University of Freiburg, 79110 Freiburg, Germany

**Keywords:** human neocortex, human dendritic spines, excitatory synapses

## Abstract

The interplay between neuronal structure and function underpins the dynamic nature of neocortical networks. Despite extensive studies in animal models, our understanding of structure–function interrelations in the adult human brain remains incomplete. Recent methodological advances have facilitated the functional analysis of individual neurons within the human neocortex, providing a new understanding of fundamental brain processes. However, the factors contributing to patient-specific neuronal properties have not been thoroughly explored. In this observational study, we investigated the structural and functional variability of superficial pyramidal neurons in the adult human neocortex. Using whole-cell patch-clamp recordings and *post hoc* analyses of dendritic spine morphology in acute neocortical slice preparations from surgical resections of seven patients, we assessed age-related effects on excitatory neurotransmission, membrane properties and dendritic spine morphologies. These results specify age as an endogenous factor that might affect the structural and functional properties of superficial pyramidal neurons.

## Teaser

Lenz *et al*. present structural and functional investigations of superficial pyramidal neurons in the adult human neocortex using neurosurgical resections. Their findings highlight the impact of age on excitatory neurotransmission, membrane properties and dendritic spines, emphasizing confounding factors in single-cell human brain investigations.

## Graphical Abstract

**Graphical Abstract:**
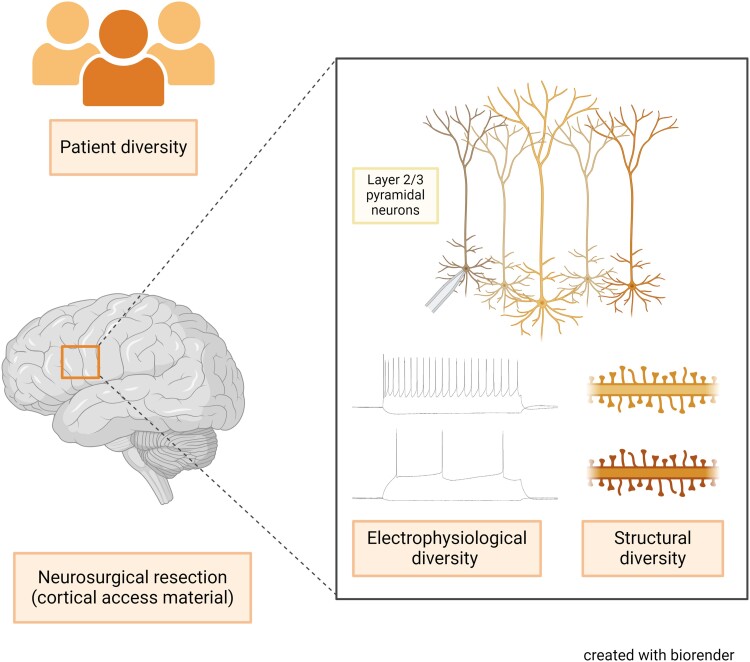

See A. Booker (https://doi.org/10.1093/braincomms/fcae366) for a scientific commentary on this article.

## Introduction

The human brain is a highly complex organ, dependent on the structural and functional properties of neurons.^1^ These neurons are continuously shaped by internal and external stimuli, allowing for adaptation to new environments, learning and memory formation.^2,3^ Although these adaptive processes are well studied in various animal and cell culture models, our understanding of human neocortical circuits remains limited.

Recent scientific progress has enhanced research of these circuits.^4,5^ Utilizing acute slice preparations from human neocortical resections, obtained during tumour or epilepsy surgery, both structural and functional properties of individual neurons can be investigated. This research has highlighted the specialization of the human neocortex in synaptic transmission, connectivity^6-8^ and signal integration,^9^ revealing that despite these unique characteristics, many adaptive processes are conserved across species.^10,11^ Nevertheless, the heterogeneity among patients presents a considerable challenge, as it may affect study outcomes and influence the conclusions that are drawn. Several attempts have been made to correlate patient characteristics—specifically age—with physiology,^12,13^ as well as neuronal dendrites and dendritic spines.^14,15^

In this study, we systematically investigated excitatory neurotransmission, intrinsic cellular properties and dendritic spine morphologies of superficial pyramidal neurons in acute slice preparations from neocortical resections of seven patients. By correlating patient metrics with single-cell analyses, we identified age-related changes in dendritic spines and we examined disease/medication conditions, specifically epilepsy/antiepileptic drugs (AEDs) and tumour/steroids, as another biological factor that could have an impact on structural and functional properties of synapses. These results highlight age as a key factor in patient heterogeneity that influences distinct synaptic features, which should be considered in future research on human neocortical resections.

## Materials and methods

### Ethics statement

Human tissue was obtained from the local biobank of the Department for Neurosurgery at the Faculty of Medicine, University of Freiburg (AZ 472/15_160880). All experiments were approved by the Local Ethics Committee, University of Freiburg (AZ 593/19). Patients gave their informed consent to use the neurosurgical resection material for research purposes. Seven samples were analysed in this observational study.

### Preparation of acute human cortical slices

Cortical resections for therapeutic or procedural indications, i.e. cortical access tissue, were performed as previously described.^16^ No macroscopic pathologies were observed in any tissue samples. Cortical tissue was transferred to chilled and carbo-oxygenated NMDG-aCSF^11^ containing (in mM) 92 NMDG, 2.5 KCl, 1.25 NaH_2_PO_4_, 30 NaHCO_3_, 20 HEPES, 25 glucose, 2 thiourea, 5 Na-ascorbate, 3 Na-pyruvate, 0.5 CaCl_2_ and 10 MgSO_4_, (pH = 7.3–7.4). Subsequently, it was embedded in low-melting-point agarose and 400 µm sections were cut with a Leica VT1200S vibratome perpendicular to the pial surface in chilled NMDG-aCSF. Subsequently, slices were transferred to cell strainers with 40 µm pores in NMDG-aCSF at 34°C. Sodium levels were gradually increased as previously described and validated.^5^ After recovery, slices were maintained in an extracellular solution containing (in mM) 92 NaCl, 2.5 KCl, 1.25 NaH_2_PO_4_, 30 NaHCO_3_, 20 HEPES, 25 glucose, 2 thiourea, 5 Na-ascorbate, 3 Na-pyruvate, 2 CaCl_2_ and 2 MgSO_4_ (pH = 7.3–7.4) at room temperature until further experimental assessment. At all times, the used medium was oxygenated continuously (5% CO_2_/95% O_2_).

### Whole-cell patch-clamp recordings

Whole-cell patch-clamp recordings were performed in a bath solution containing (in mM) 92 NaCl, 2.5 KCl, 1.25 NaH_2_PO_4_, 30 NaHCO_3_, 20 HEPES, 25 glucose, 2 thiourea, 5 Na-ascorbate, 3 Na-pyruvate, 2 CaCl_2_ and 2 MgSO_4_ (pH = 7.3–7.4). Recordings were carried out at 35° under continuous oxygenation (5% CO_2_/95% O_2_) and three to six cells were patched per slice. Cells were visually identified using an LN-Scope (Luigs and Neumann, Germany) equipped with infrared Dodt gradient contrast and a ×40 water-immersion objective (numerical aperture 0.8; Olympus). Human superficial (layer 2/3) pyramidal cells were visualized on the pia-white matter axis. The median distance from the pial surface was 670 µm (25–75% percentile: 570.5–773.0 µm). Electrophysiological signals were amplified using a Multiclamp 700B amplifier, digitized with a Digidata 1550B digitizer and visualized with the pClamp 11 software package. Patch pipettes contained (in mM) 126 K-gluconate, 4 KCl, 10 HEPES, 4 MgATP, 0.3 Na_2_GTP, 10 PO-creatine and 0.3% (*w*/*v*) biocytin (pH = 7.25 with KOH; 285 mOsm/kg) and had a tip resistance of 3–5 MΩ. Cells were recorded in voltage-clamp mode at a holding potential of −70 mV. Intrinsic cellular properties were recorded in current-clamp mode following spontaneous excitatory postsynaptic current (sEPSC) recordings. Pipette capacitance of 2.0 pF was corrected and series resistance was compensated using the automated bridge balance tool of the MultiClamp commander. I-V curves were generated by injecting 1 s square pulse currents starting at −100 pA and increasing in 10 pA steps until +1100 pA current injection was reached with a sweep duration of 2 s. Series resistance was monitored before and after recording, and recordings were discarded if the resistance reached ≥30 MΩ. Additionally, cells were excluded from further analyses if series resistance changed >5 MΩ during recordings [median Rs before recording: 11.80 MΩ (9.448–14.04 MΩ; 25–75% percentile); median Rs after recording: 11.86 MΩ (10.23–13.84 MΩ; 25–75% percentile); median change over recording time: 6.32%]. The liquid junction potential was calculated to be 15.2 mV and was not corrected.

### Immunofluorescence and *post hoc* staining

After electrophysiological assessment, cortical slices were fixed in 4% (*w*/*v*) paraformaldehyde [in phosphate buffered saline (PBS; 0.1 M, pH 7.4) with 4% (*w*/*v*) sucrose] overnight. After fixation, they were washed in PBS (0.1 M, pH 7.4) and incubated with 10% (*v*/*v*) normal goat serum in 0.5% (*v*/*v*) Triton X-100 containing PBS for 1 h, in order to reduce nonspecific staining. For immunofluorescence, slices were incubated with an anti-NeuN antibody [rabbit polyclonal, 1:1000 (1 µg/mL); #ab104225, Abcam] in 10% (*v*/*v*) normal goat serum in 0.1% (*v*/*v*) Triton X-100 containing PBS at 4°C overnight. After washing, suitable secondary antibodies [goat anti-rabbit, Alexa Fluor Plus 555, 1:1000 (2 µg/mL); #A-32732, Invitrogen] were added during the *post hoc* visualization of patched neurons, for which the tissue was incubated with Streptavidin Alexa Fluor 488 [Streptavidin A488, 1:1000 (2 µg/mL); #S32354, Invitrogen] diluted in 10% (*v*/*v*) normal goat serum in 0.1% (*v*/*v*) Triton X-100 containing PBS at 4°C overnight. After washing, the tissue was incubated with DAPI nuclear stain [1:5000 (0.2 µg/mL) in PBS for 15 min; #62248, Thermo Scientific] in order to visualize cytoarchitecture, transferred onto glass slides and mounted with a fluorescence anti-fading mounting medium (DAKO Fluoromount; #S302380-2, Agilent). In order to reduce age-related autofluorescence, slices were incubated with Sudan black [0.1% (*w*/*v*) in 70% ethanol] for 10 min before the DAPI staining.

### Confocal imaging

For visualization, confocal images were acquired using a Leica SP8 laser-scanning microscope equipped with a ×20 multi-immersion (numerical aperture 0.75; Leica), a ×40 oil-immersion (numerical aperture 1.30; Leica), and a ×63 oil-immersion objective (numerical aperture 1.40; Leica). For structural analysis of dendritic spines, an overview image of each cell was generated with the ×40 oil-immersion objective setting the limits of the *z*-axis ∼10 µm in each direction from the middle of the soma (*z*-step: 0.6 µm). Apical and basal dendritic segments were randomly chosen and their position tagged in the overview image. Five to 25 segments of each cell were recorded using the ×63 oil-immersion objective (immersion oil refractive index: 1.52, resolution: 2048 × 2048 pixels, zoom: ×4, speed: 200 Hz, pinhole: 0.6 AU, *z*-step: 0.1 µm; calculated pixel size: 23 nm) without deconvolution. Laser intensity and detector gain were set individually, in order to achieve comparable overall fluorescence intensities throughout stacks. Confocal image stacks were stored as .tif files.

### Quantification and statistics

Electrophysiological data were assessed using the pClamp 11 software package (Axon Instruments). sEPSC properties were analysed with an automated template-based search tool for event detection. The template was previously created in the Clampfit11 software based on manually identified and selected EPSCs. The template match threshold was set at 2.5, and the time period for the allowed duration of a single event starting from the baseline was defined. Both parameters were not changed within the different recordings or groups. Resting membrane potential was assessed from the baseline value of the I-V curve. Input resistance was calculated for the injection of −100 pA current at a time frame of 200 ms at the end of the current step.

Structural analysis of spine density and volume was conducted by investigators blinded to experimental conditions. Spine density was assessed by counting spines manually in the *z*-stack of the confocal images with the ImageJ software package (https://imagej.net) and normalizing the number to the length of the segment. Spine volume was analysed using the surface detection tool of the Imaris 9.5 software package (Oxford Instruments).

Data were statistically analysed using GraphPad Prism 10 (GraphPad Software, USA). Seven samples have been included in this study, each with completed electrophysiological and morphological dendritic spine analyses (for overview, see Table 1). Results from electrophysiological (individual cells) and morphological analyses (individual dendritic segments) were documented for each sample, with further analyses performed using averaged data (both mean and median) from each patient. The correlation matrix for continuous sample data and corresponding *P*-value calculations were performed with the Spearman *r* correlation using mean values and were depicted as a heatmap. Moreover, we performed linear regression analysis. *P*-values for detecting non-zero slope and *R*^2^ values for the goodness of fit are provided in figures along with Spearman *r* for correlation analysis. For group comparisons, we used a nested *t*-test. *P*-values <0.05 were considered statistically significant (*P < 0.05, **P < 0.01, and ***P < 0.001); results without statistical significance were indicated as ‘ns’. All violin plots depict the median with 25–75% percentiles, and all linear regression analysis graphs depict median ± interquartile range. In the violin plots, individual values are indicated by coloured dots, with the median illustrated as a black line. A number of replicates in patient samples are provided in the figure legends. Source files for the figured graphs were provided as supplementary materials.

**Table 1:** Summary of patient data and combined results from functional and structural cellular assessments

#	Age	Sex	Epilepsy	AED medication	Tumour	Steroid medication	Region	sEPSC amplitude [pA]	sEPSC frequency [Hz]	Input resistance [MΩ]	Resting membrane potential [mV]	AP frequency [Hz; 600 pA injection]	Spine head volume [µm^3^]	Dendritic spine density [count/µm]	Distance to pia mater [µm, range of values]
**1**	39	Female	Yes	Yes	No	No	Temporal	21.56 [20.00–24.62; 15]	1.351 [1.080–1.792; 15]	66.61 [52.40–101.5; 14]	−65.05 [−71.17 to −59.53; 14]	18.5 [14.25–22.5; 14]	0.1233 [0.1002–0.1422; 76]	1.201 [0.971–1.434; 76]	495–825
**2**	18	Female	No	No	Yes	Yes	Temporal	21.07 [19.01–23.28; 35]	1.187 [0.994–1.834; 35]	40.98 [35.27–48.16; 35]	−73.02 [−74.96 to −69.05; 35]	5.0 [1–7; 35]	0.1223 [0.1051–0.1427; 116]	1.851 [1.532–2.191; 117]	545–677
**3**	27	Female	Yes	Yes	No	No	Temporal	24.59 [21.14–27.24; 21]	3.022 [1.959–3.569; 21]	42.34 [36.61–67.96; 21]	−68.79 [−75.20 to −66.72; 21]	10.0 [5–14.5; 21]	0.1174 [0.0956–0.1403; 39]	1.337 [1.050–1.598; 39]	475–900
**4**	78	Female	No	No	Yes	Yes	Frontal	22.27 [20.73–22.69; 19]	1.376 [1.071–2.633; 19]	51.87 [41.02–74.80; 19]	−66.61 [−69.78–−62.07; 19]	14.0 [2–22; 19]	0.0932 [0.0799–0.1443; 27]	0.8582 [0.703–1.100; 27]	960–1160
**5**	54	Male	No	No	Yes	Yes	Temporal	24.03 [20.66–25.89; 23]	2.687 [1.822–3.666; 23]	42.81 [36.85–51.60; 23]	−69.21 [−71.35 to −67.33; 23]	12.0 [8–14; 23]	0.1097 [0.0919–0.1375; 60]	1.023 [0.818–1.251; 60]	605–840
**6**	55	Male	Yes	Yes	No	No	Temporal	22.29 [20.94–23.49; 14]	2.463 [1.384–3.369; 14]	52.46 [43.00–66.80; 14]	−67.18 [−69.05 to −62.94; 14]	14.0 [10–22; 14]	0.1522 [0.1187–0.1705; 30]	1.159 [0.963–1.282; 30]	536–700
**7**	52	Female	Yes	Yes	No	No	Temporal	23.75 [22.88–26.12; 29]	2.473 [1.557–4.012; 29]	39.92 [29.22–48.59; 29]	−61.59 [−68.51 to −59.39; 29]	8.0 [4–12; 29]	0.1542 [0.1264–0.1741; 53]	0.9118 [0.781–1.069; 53]	635–1135

For continuous data, median [25–75% percentiles; number of cells or dendritic segments] was reported in each sample.

### Digital illustrations

Confocal images were stored as .tif files, and image brightness and contrast were adjusted. Figures were prepared using the ImageJ software package (https://imagej.net) and Photoshop graphics software (Adobe, San Jose, CA, USA).

## Results

### Correlation analysis of patient metrics with structural and functional properties of superficial pyramidal neurons reveals age-dependent changes in dendritic spine density

**Figure 1:**
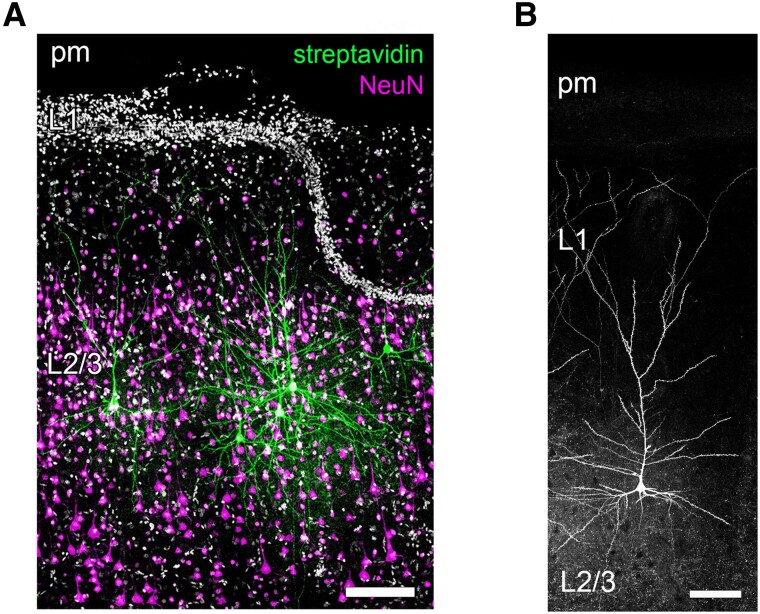
**Superficial pyramidal neurons in the human neocortex.** (**A**) Representative image of *post hoc* stained human cortical superficial pyramidal neurons (streptavidin) with neuronal cell bodies marked by NeuN staining. L1, layer 1; L2/3, layer 2/3; pm, pia mater. Scale bar: 150 µm. (**B**) *Post hoc* stained superficial pyramidal neuron in the human cortex. L1, layer 1; L2/3, layer 2/3; pm, pia mater. Scale bar: 100 µm.

This study included human neocortical resections from seven patients. We analysed structural and functional properties of superficial pyramidal neurons in acute slice preparations. Within these preparations, cortical lamination and the dendritic tree of principal cells are preserved (Fig. 1A and B). We correlated continuous (e.g. age) and categorical (sex, resection locus and disease entity) patient data with single-cell electrophysiological recordings and morphological assessments of *post hoc*-visualized neurons (Table 1). The patient cohort consisted of individuals aged 18–78 years, including two males and five females. Three patients had tumour resections, and four underwent epilepsy surgery, with treatments involving either steroid (short-term and intra-operative) or antiepileptic medication.

**Figure 2:**
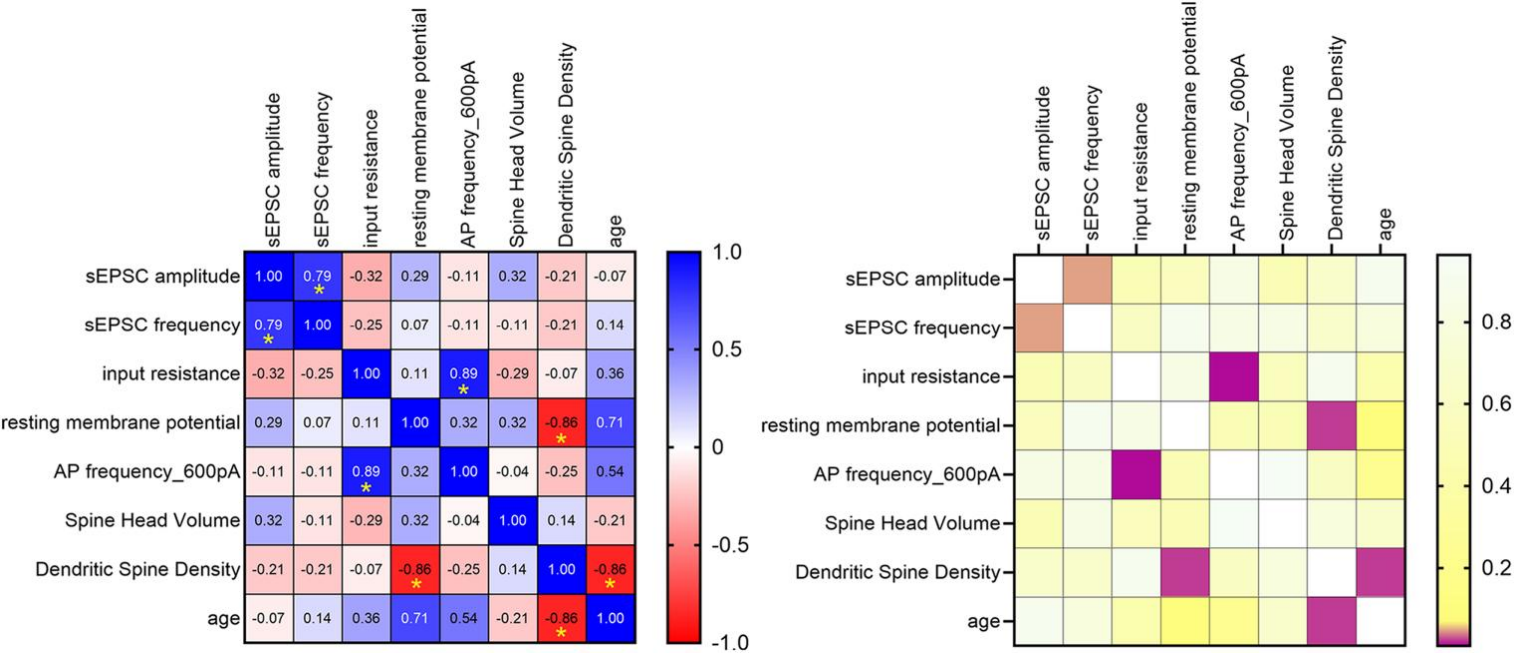
**Correlation of structural and functional properties of superficial human pyramidal neurons with clinical patient parameters.** Correlation matrix displaying Spearman correlations of continuous numerical data (mean values, left panel) and corresponding *P*-values (right). Significant results are highlighted with asterisks (left panel) and violet squares (right panel), showing correlations between synaptic and electrophysiological parameters, and between patient age and dendritic spine density.

**Figure 3:**
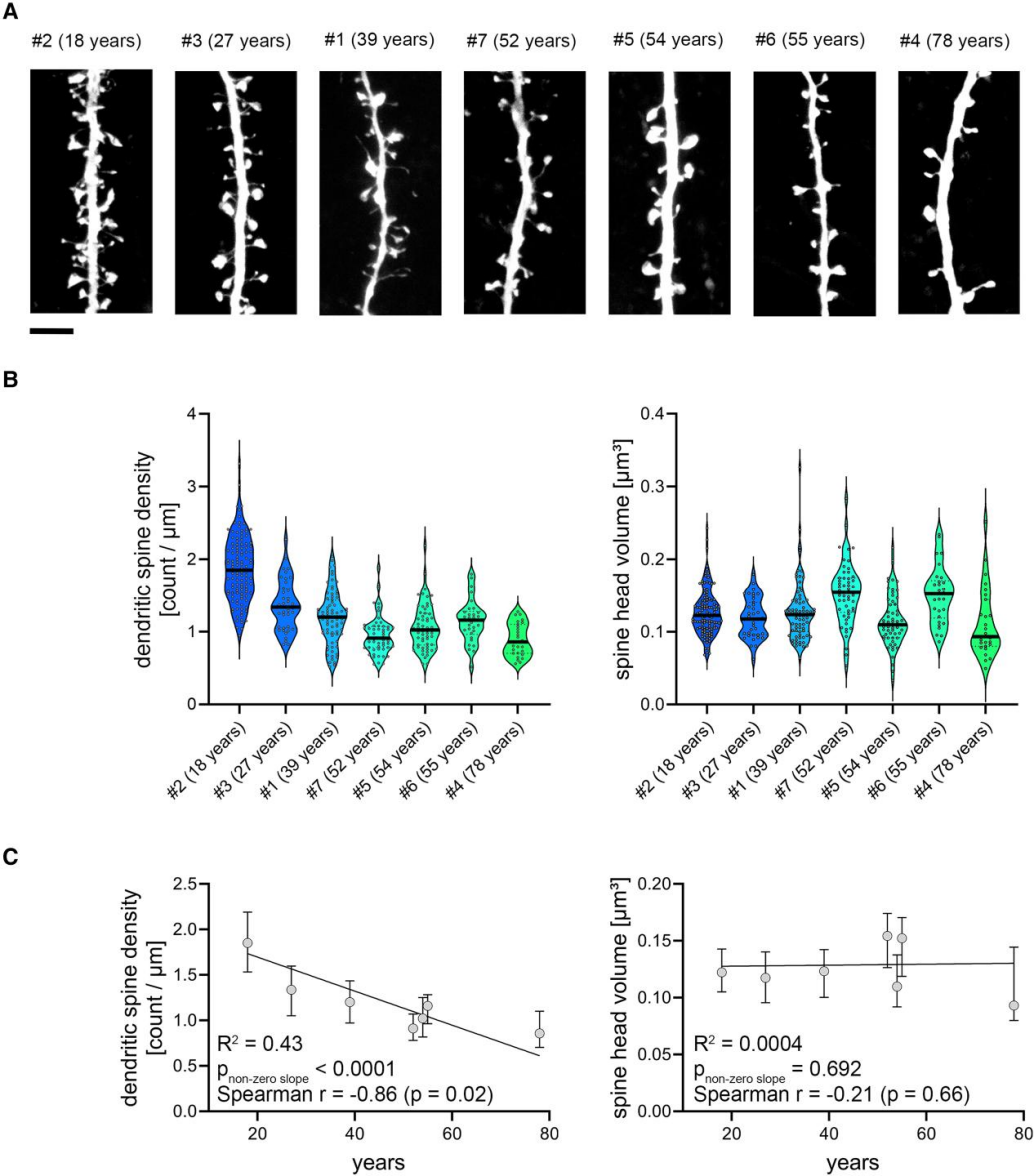
**Structural analysis of dendritic spine morphology in human superficial pyramidal neurons.** (**A**) Example images of *post hoc* labelled dendritic segments with spines (streptavidin staining) from superficial pyramidal neurons, sampled from seven patients. Scale bar: 4 µm. (**B**) Left panel: Analysis of dendritic spine density [#2 (18 years) = 117 segments in six cells; #3 (27 years) = 39 segments in five cells; #1 (39 years) = 76 dendritic segments in six cells; #7 (52 years) = 53 segments in five cells; #5 (54 years) = 60 segments in five cells; #6 (55 years) = 30 segments in three cells; #4 (78 years) = 27 segments in three cells]. Right panel: analysis of dendritic spine head volumes [c.f. Fig. 2; #2 (18 years) = 116 segments in six cells; #3 (27 years) = 39 segments in five cells; #1 (39 years) = 76 dendritic segments in six cells; #7 (52 years) = 53 segments in five cells; #5 (54 years) = 60 segments in five cells; #6 (55 years) = 30 segments in three cells; #4 (78 years) = 27 segments in three cells]. Individual mean values from dendritic segments are indicated by grey dots. (**C**) Left panel: *X*-*Y* graph displaying the correlation between age and overall dendritic spine density across various age groups from the same data as shown in **B**. A linear regression is shown as black line. Right panel: *X*-*Y* graph illustrating the lack of significant correlation between age and overall dendritic spine head volume for the same age groups and segments counts. A linear regression is indicated with a black line (*P*-values for non-zero slope and *R*^2^ values for goodness of fit are reported in the figure, along with Spearman *r* for correlation analysis). All violin plots depict the median with 25–75% percentiles, individual values are indicated by coloured dots, and the median is illustrated as a black line. All linear regression analysis graphs depict median ± interquartile range.

First, we performed correlative analyses between continuous patient data and the following electrophysiological parameters: AMPA receptor-mediated sEPSCs [sEPSC amplitude (pA) and frequency (Hz)], intrinsic cellular properties [input resistance (MΩ), resting membrane potential (mV) and action potential frequency (Hz)] and dendritic spine metrics [spine head volume (µm^3^) and dendritic spine density (count per µm); Fig. 2; statistical assessment using mean values for correlation analysis summarized in Supplementary Table 1]. Our findings included (i) a positive correlation between sEPSC amplitude and frequency (*R*^2^ = 0.71, *P*_non-zero slope_ = 0.017, Spearman *r* = 0.79; *P* = 0.048), (ii) a positive correlation between action potential frequency and input resistance (*R*^2^ = 0.75, *P*_non-zero slope_ = 0.012, Spearman *r* = 0.89; *P* = 0.012), (iii) a negative correlation between dendritic spine density and resting membrane potential (*R*^2^ = 0.72, *P*_non-zero slope_ = 0.015, Spearman *r* = −0.86; *P* = 0.024) and (iv) a negative correlation between dendritic spine density and patient age (*R*^2^ = 0.43, *P*_non-zero slope_ < 0.0001, Spearman *r* = −0.86; *P* = 0.024; c.f. Fig. 3C). No significant age-dependent correlation was observed with spine head volume (*R*^2^ = 0.0004, *P*_non-zero slope_ = 0.69, Spearman *r* = −0.21; *P* = 0.662; c.f. Fig. 3C).

### Dendritic spine analysis illustrates an age-related decline in dendritic spine density

We analysed spine morphology and density on various segments of both apical and basal dendrites in superficial pyramidal neurons (Fig. 3A). Our analyses illustrate individual data points for dendritic spine densities and spine head volumes across patients (Fig. 3B; source data summarized in Supplementary Table 2). Unlike the age-dependent decrease in dendritic spine densities, no age-dependent correlations were found for spine head volumes (Fig. 3C; statistical assessment in Supplementary Table 2).

### Electrophysiological assessment demonstrates stability of excitatory neurotransmission in aging

**Figure 4:**
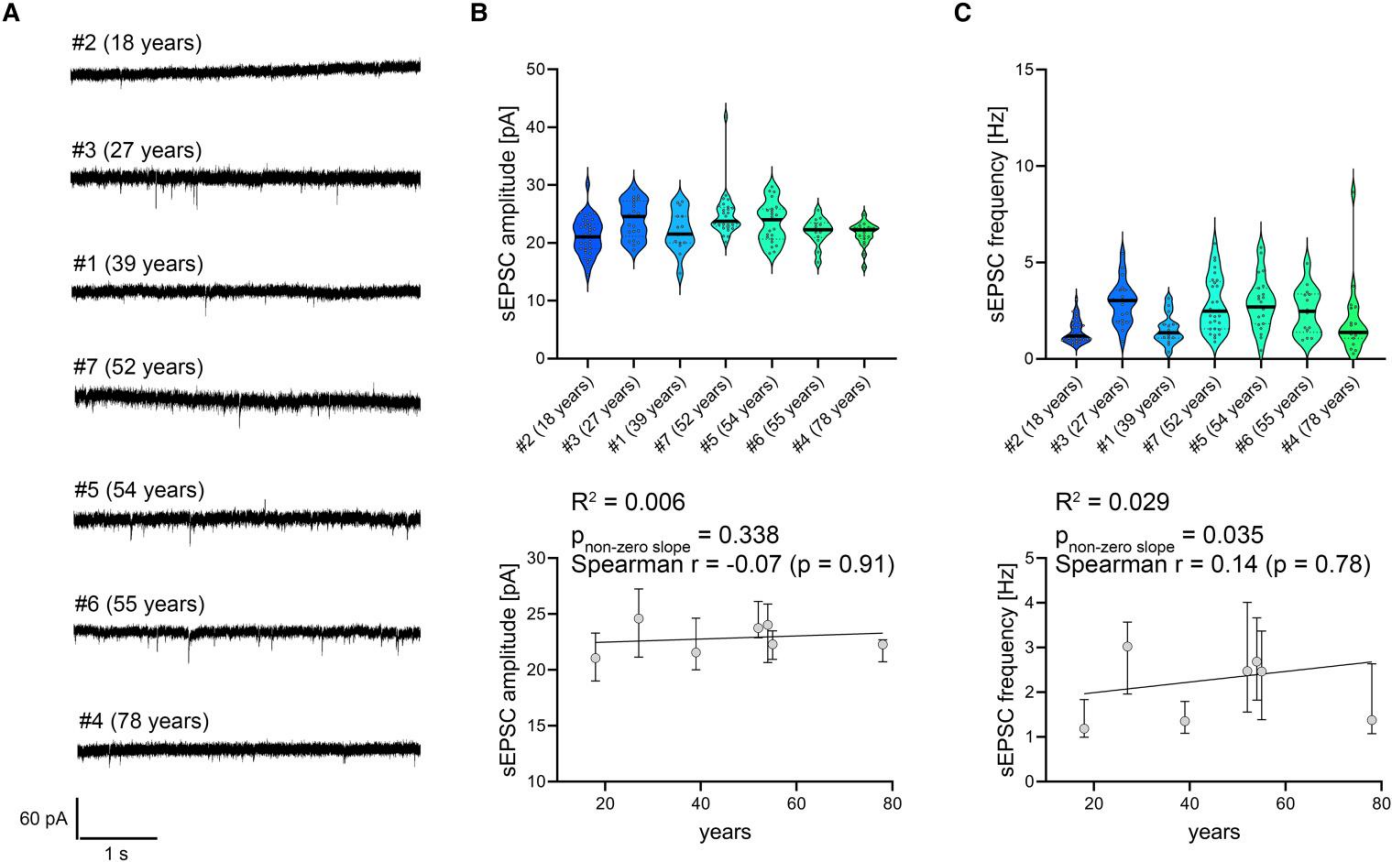
**Electrophysiological assessment of excitatory synaptic transmission in human superficial pyramidal neurons.** (**A**) Sample traces of AMPA receptor-mediated sEPSCs in superficial pyramidal neurons from seven adult human neocortex samples (sorted by age). (**B**, **C**) Quantitative analysis of sEPSC amplitudes (**B**) and frequencies (**C**), indicating the absence of age-related changes [#2 (18 years) = 35 cells; #3 (27 years) = 21 cells; #1 (39 years) = 15 cells; #7 (52 years) = 29 cells; #5 (54 years) = 23 cells; #6 (55 years) = 14 cells; #4 (78 years) = 19 cells]. Grey dots represent individual cell values within each sample. *X*-*Y* graphs demonstrating the respective median ± interquartile range across different ages. A linear regression is indicated with a black line (*P*-values for non-zero slope and *R*^2^ values for goodness of fit are reported in the figure, along with Spearman *r* for correlation analysis). All violin plots depict the median with 25–75% percentiles, individual values are indicated by coloured dots, and the median is illustrated by a black line. All linear regression analysis graphs depict median ± interquartile range.

For functional assessments, we conducted whole-cell patch-clamp recordings of sEPSCs from superficial pyramidal neurons in acute slice preparations of the adult human neocortex (Fig. 4A). Our results illustrate the heterogeneity of cells in both sEPSC amplitudes and frequencies in individual patient samples. Here, no significant age-dependent correlations were found (Fig. 4B and C; source data and statistical assessment in Supplementary Table 3).

**Figure 5:**
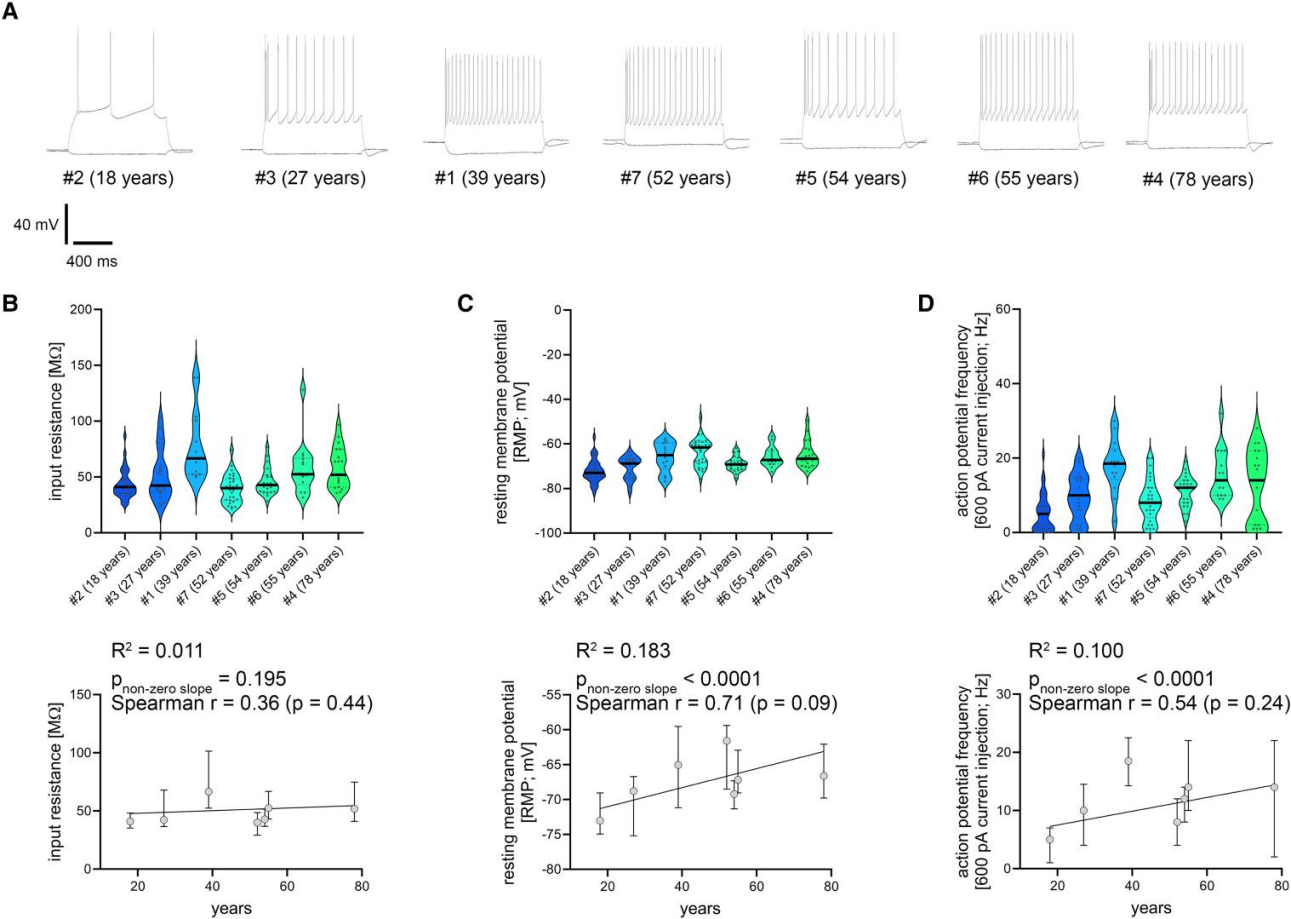
**Electrophysiological assessment of intrinsic membrane properties in human superficial pyramidal neurons.** (**A**) Representative current clamp input–output curve traces depicting responses at −100 and +600 pA current injections in superficial pyramidal neurons. (**B–D**) Upper panels: analysis of passive [input resistance (**B**) and resting membrane potential (**C**)] and active (**D**; action potential frequency) membrane properties [#2 (18 years) = 35 cells; #3 (27 years) = 21 cells; #1 (39 years) = 14 cells (one cell excluded from analysis, since integrity of the patch was lost during the recordings); #7 (52 years) = 29 cells; #5 (54 years) = 23 cells; #6 (55 years) = 14 cells; #4 (78 years) = 19 cells]. Grey dots represent individual cell values within each sample. Lower panels: *X*-*Y* graphs demonstrating the respective median ± interquartile range across different ages. A linear regression is indicated with a black line (*P*-values for non-zero slope and *R*^2^ values for goodness of fit are reported in the figure, along with Spearman *r* for correlation analysis). All violin plots depict the median with 25–75% percentiles, individual values are indicated by coloured dots, and the median is illustrated by a black line. All linear regression analysis graphs depict median ± interquartile range.

Additionally, both passive (resting membrane potential and input resistance) and active membrane properties (action potential frequencies) were evaluated using input–output curve recordings (Fig. 5A). While there was no significant correlation between age and either input resistance (Fig. 5B; *P* = 0.44) or action potential frequencies (Fig. 5D; *P* = 0.24), we observed a non-significant trend towards an age-related increase in resting membrane potential (Fig. 5C; *P* = 0.09). In conclusion, our structural and functional assessments of superficial pyramidal neurons in the adult human neocortex highlight age-dependent changes of synaptic properties.

### Influence of disease/treatment on structural and functional properties of superficial pyramidal neurons

In further comparative analyses, we examined whether the underlying disease and associated treatment might affect the structural and functional properties of superficial pyramidal neuron synapses in the human neocortex. We defined two patient groups: Group 1, diagnosed with epilepsy/seizures and treated with AEDs (epilepsy/AED), and Group 2, diagnosed with a tumour and treated with steroids (tumour/steroids). In this cohort, epilepsy patients did not receive steroids and tumour patients did not receive antiepileptic medication due to the lack of symptomatic seizures (c.f. Table 1).

**Figure 6:**
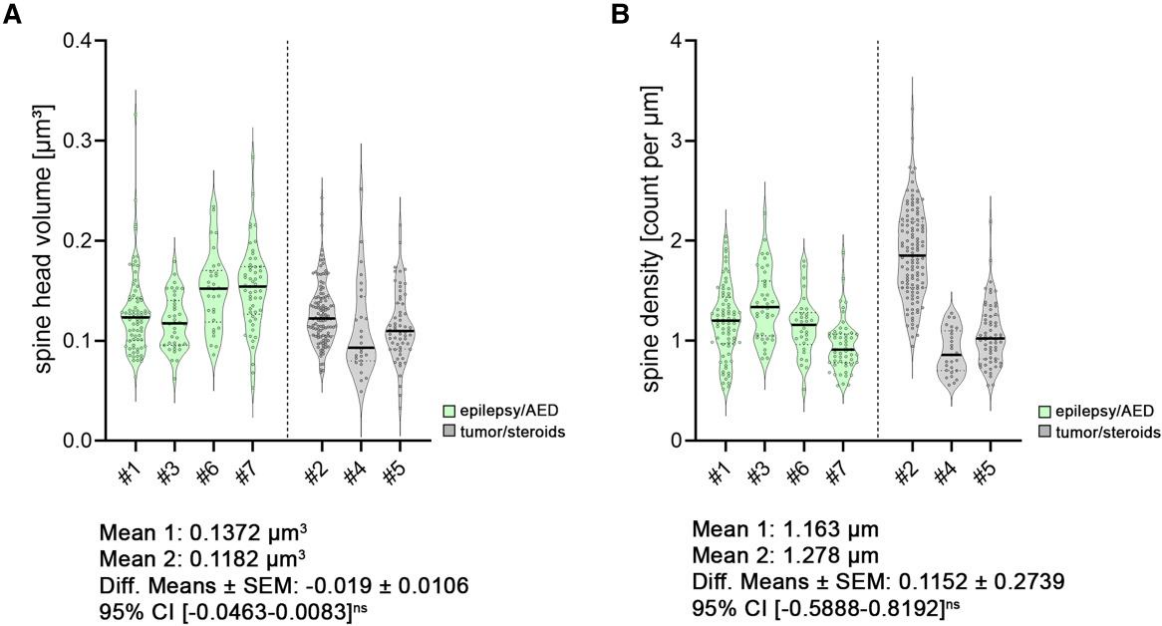
**Disease and treatment related changes in structural properties of superficial pyramidal neurons.** Results classified by medical records into two groups: epilepsy/antiepileptic medication (AED) and tumour/steroid medication (c.f. Table 1). (**A**) Group data of spine head volumes in dendritic compartments (A; *n*_epilepsy/AED_ = 198 segments in four patients; *n*_tumour/steroids_ = 203 segments in three patients; c.f. Table 1; nested *t*-test). (**B**) Group data of dendritic spine densities (**A**; *n*_epilepsy/AED_ = 198 segments in four patients, *n*_tumour/steroids_ = 204 segments in three patients; c.f. Table 1; nested *t*-test). All violin plots depict the median with 25–75% percentiles, individual values are indicated by coloured dots, and the median is illustrated as a black line. A nested *t*-test was used for group comparisons. *P*-values <0.05 were considered statistically significant; results without statistical significance were indicated as ‘ns’.

**Figure 7:**
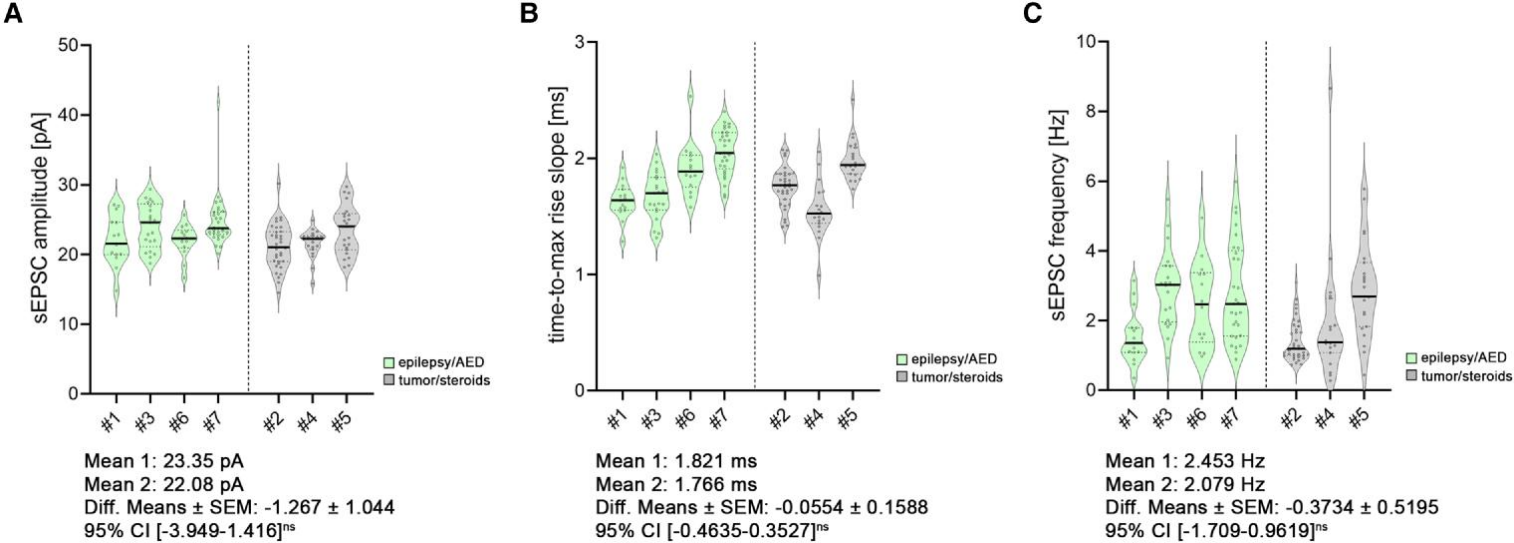
**Disease and treatment related changes in excitatory synaptic transmission of superficial pyramidal neurons.** (**A–C**) Group data of AMPA receptor-mediated sEPSC amplitudes (**A**), time-to-max rise slope (**B**) and sEPSC frequencies (**C**) (*n*_epilepsy/AED_ = 79 cells in four patients; *n*_tumour/steroids_ = 77 cells in three patients; nested *t*-test). All violin plots depict the median with 25–75% percentiles, individual values are indicated by coloured dots, and the median is illustrated as a black line. A nested *t*-test was used for group comparisons. *P*-values <0.05 were considered statistically significant; results without statistical significance were indicated as ‘ns’.

**Figure 8:**
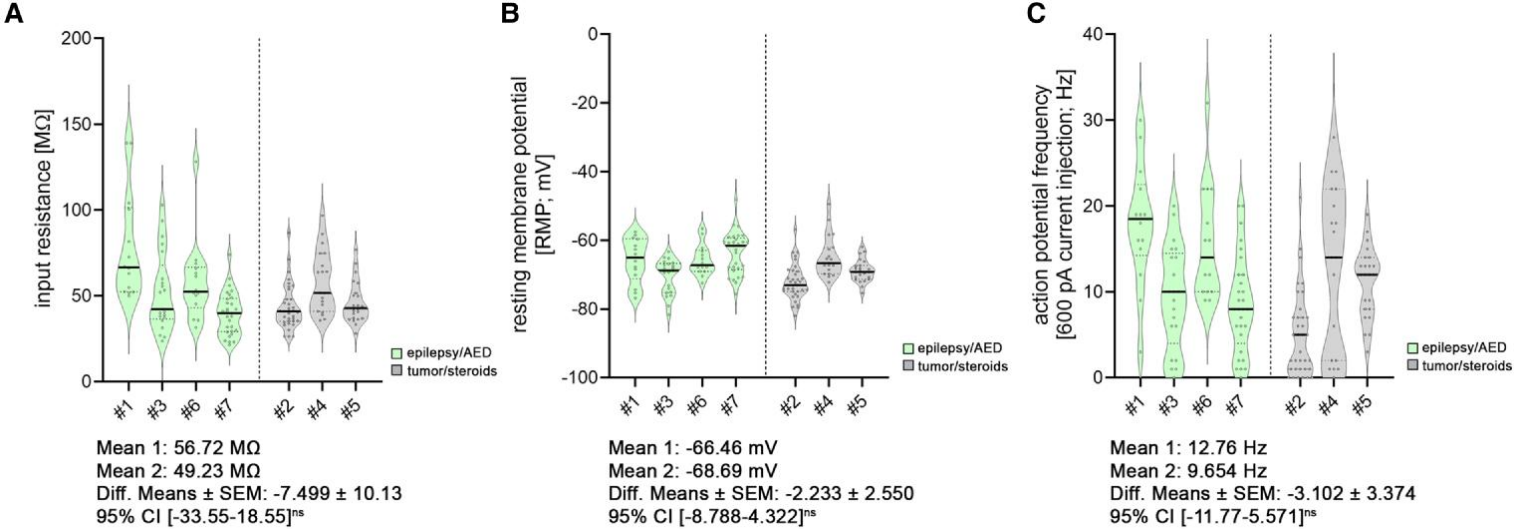
**Disease and treatment related changes in intrinsic cellular properties of superficial pyramidal neurons.** (**A–C**) Analysis of passive (**A**; input resistance; **B**; resting membrane potential) and active (**C**; action potential frequency) membrane properties [*n*_epilepsy/AED_ = 78 cells in four patients (one cell excluded from further analysis due to a decline in patch integrity); *n*_tumor/steroids_ = 77 cells in 3 patients; nested *t*-test]. All violin plots depict the median with 25–75% percentiles, individual values are indicated by coloured dots, and the median is illustrated as a black line. A nested *t*-test was used for group comparisons. *P*-values <0.05 were considered statistically significant; results without statistical significance were indicated as ‘ns’.

We compared cellular properties between these groups [Figs 6–8; statistical assessment with averaged patient data in Supplementary Table 4 (spine analysis) and Supplementary Table 5 (electrophysiological analysis)]. Although we identified some trends in this comparatively small cohort, no significant differences related to disease entity or medication were detectable. (i) No changes in spine head volumes were detected (*P* = 0.13, *F* = 3.187; nested *t*-test; Fig. 6A). (ii) No differences in dendritic spine densities were detected (*P* = 0.69, *F* = 0.177; nested *t*-test; Fig. 6B). Of note, detailed analyses in either apical or basal dendritic segments did not show compartmental differences (apical: spine head volume, *P* = 0.08, *F* = 4.972, nested *t*-test; dendritic spine density, *P* = 0.55, *F* = 0.402, nested *t*-test; basal: spine head volume, *P* = 0.3, *F* = 1.333, nested *t*-test; dendritic spine density, *P* = 0.84, *F* = 0.047, nested *t*-test). (iii) The epilepsy/AED group showed no significant difference in sEPSC amplitudes (*P* = 0.28, *F* = 1.473; nested *t*-test; Fig. 7A), which was also the case for the time-to-max rise slope (*P* = 0.74, *F* = 0.122; nested *t*-test; Fig. 7B) and frequencies (*P* = 0.50, *F* = 0.517; nested *t*-test; Fig. 7C). (iv) Moreover, intrinsic membrane properties, i.e. input resistance (*P* = 0.49, *F* = 0.548; nested *t*-test; Fig. 8A), resting membrane potential (*P* = 0.42, *F* = 0.767; nested *t*-test; Fig. 8B) and action potential frequency (*P* = 0.40, *F* = 0.845; nested *t*-test; Fig. 8C), were indistinguishable.

## Discussion

This study investigated excitatory neurotransmission, intrinsic cellular properties and dendritic spines of superficial pyramidal neurons in seven patients, assessing both structural and functional parameters. By correlating patient metrics with single-cell data, we observed an age-dependent decrease in dendritic spine densities, while other spine parameters and functional properties remained unaffected by age. We further examined the influence of disease/medication on distinct cellular properties: although we observed trends that should be re-addressed in larger patient cohorts, no significant influence of disease/medication conditions was detected. However, these results underscore the impact of heterogeneity in human cohorts, which must be considered in research on human neocortical resections.

Recent studies have described a prominent supragranular expansion in the evolution of the human neocortex, which is accompanied by a diversification of glutamatergic neurons.^17,18^ This diversity is evident in multiple parameters, such as h-current, frequency preferences and dendrite properties.^18,19^ Some parameters depend on the depth from the pia, while others appear to be independent.^19^ In our study, variance in functional and structural data was also evident within individual samples. Therefore, cellular heterogeneity must be considered a confounding factor in our correlative analyses. Moreover, it is interesting to speculate whether the diversity of glutamatergic neurons changes during ageing.

The central nervous system is distinguished by its ability to adapt structurally and functionally to various stimuli, subsumed by the term ‘plasticity’.^20^ Pathological conditions noted in medical records or patient metrics, such as seizures or AEDs, likely trigger adaptive processes in neural networks.^21,22^ These adaptations enable appropriate responses to external stimuli, are crucial for behavioural flexibility, learning and memory, and are maintained by the expression of homeostatic plasticity.^23^ Originally defined as a negative feedback mechanism regulating all synapses of a neuron (referred to as synaptic scaling^24^), substantial evidence now suggests that homoeostatic synaptic plasticity can also occur locally in specific sets of synapses.^25-27^ Although the biological importance of homeostatic plasticity is still under debate, its involvement in memory specificity and acquisition has been increasingly acknowledged.^28^ It is interesting to consider how age-related changes may reflect differential recruitment of neural plasticity mechanisms.

Age-related cognitive decline is a common phenomenon that significantly contributes to healthcare burden.^29-31^ A central finding of our study is the negative correlation between age and dendritic spine density, suggesting a structural correlate of ageing in dendritic trees of superficial pyramidal neurons, akin to observations in mice and rats^32-34^ and previous reports in humans.^35^ A major advantage of morphological analysis of dendritic spines is its independence from functional dendritic parameters that might influence whole-cell patch-clamp recordings at the soma due to dendritic attenuation.^36^ Moreover, distinct rules in synapse reliability and spine-to-dendrite coupling might be relevant in the human brain.^37-40^ It is important to note that our data were obtained using conventional confocal microscopy, which does not achieve the resolution of super-resolution microscopy techniques (e.g. stimulated emission depletion^41^). The assessment of spine head volumes might be affected by the diffraction limit of light. Although reported volumes might not reflect the exact size of dendritic spines, comparison between samples remains valid. Moreover, our findings on spine density do not rely on high-precision volume analyses. Other parameters, such as excitatory neurotransmission, appeared largely unaffected by the age of patients, indicating the activation of compensatory mechanisms at the functional level. We hypothesize that compensatory mechanisms and their potential exhaustion could contribute to age-related cognitive decline. Thus, strategies to identify and stabilize these mechanisms may offer promising therapeutic avenues.

Studies utilizing human neocortical samples face challenges due to patient heterogeneity and limited sample availability. Despite the limited number of samples in this study, we emphasize that patient metrics and medical records must be considered when interpreting experimental data. This notion is supported by reports of various medications affecting the central nervous system, as seen for example in the context of mood and personality changes associated with AEDs.^42^ Effective disease management, such as controlling epileptic seizures, often requires individual selection and adjustment of medication, adding another level of complexity.^43-47^ Given the limited number of samples in this study, we did not address regional differences, despite the well-documented morphological specialization of brain regions,^48^ nor did we consider the sex of patients. Additionally, grouping patients based on similarities was not feasible in this cohort. A larger sample size is needed to account for the numerous layers of biological and medical variability. Nevertheless, human neocortical resections provide a unique opportunity to examine the structure and function of human neural circuits.^49,50^ Although there are similarities between human and rodent brains,^11,51^ significant differences in signal integration and plasticity induction have been observed.^6-9,52,53^ Our results further confirm the presence of structural and functional plasticity in the human brain, underscoring its evolutionary preservation and significance across species and research models.

Taken together, this study highlights the crucial role of patient metrics in understanding the human neocortex. Our findings underscore the need for an integrated research approach that combines detailed clinical profiles with analyses on a single-cell level. A better grasp of these mechanisms not only deepens our understanding of the human brain but also enhances translational research.

## Supplementary Material

fcae351_Supplementary_Data

## Data Availability

Data source files and statistical evaluations are provided in the supplementary materials. The data that support the findings of this study are available from the corresponding author, upon reasonable request.
